# Challenges of agriculture-related eye injuries in Nigeria

**Published:** 2015

**Authors:** Fatima Kyari

**Affiliations:** Ophthalmologist/Senior Lecturer: College of Health Sciences, University of Abuja, Nigeria.

**Figure:**
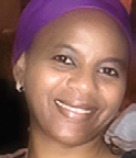
Fatima Kyari

Agriculture, which includes crop farming, livestock rearing and fishing, provides work for up to 70% of the labour force in Nigeria.^1^ The agricultural sector contributes up to 20% of the gross domestic product (GDP) of Nigeria, with an average real growth rate of 3.5% from 2014 to 2015.^2^

People involved in agriculture and farm-related activities are at greater risk of eye injuries. Unpublished data from the Nigeria National Blindness and Visual Impairment Survey showed that, of the participants who had a history of eye injury, over half (53%) were farmers. Of those without a history of eye injury, only 39% were farmers. A 5-year hospital review of people with eye injuries showed that more than two-thirds of all eye injuries were sustained on a farm. Although non-penetrating eye injuries were more common, 15% of people were already blind in the injured eye at presentation.^3^ In a multi-centre retrospective review of ocular trauma among older people, eye injury most commonly occurred on the farm (37.2%).^4^

Some of the **main causes** of agriculture-related eye injuries include:

1. Accidental direct trauma with farm implements (e.g. cutlass, hoe, fishing hook, etc.)
2. Vegetable/plant/organic material hitting the eye, or spillage into the eye (cocoa pod, cornstalks, sticks/twigs, palm tree stalks, thorn, leaf, kernel, etc.)
3. Sand spillage into eye
4. Other foreign body (FB) in the eye
5. Animal attack injury (e.g. cow horn injury, spitting cobra, insect sting)
6. Assault injuries during communal conflicts involving crop farmers and cattle herdsmen.

A hospital series reported vegetative/plant material as a cause of 42% of eye injuries.^5^ Cow horn injury is an important cause of monocular blindness as it often results in severe open globe injuries with corneoscleral lacerations.^6^,^7^ Life-threatening poisonous arrow injuries to the eye sustained during communal conflicts between farmers have also been reported.^8^

The **effects of injury to the eye** include:

1. Embedded foreign body in the eye
2. Corneal abrasion
3. Traumatic cataract
4. Penetrating laceration resulting in lens injury, vitreous haemorrhage, or retinal tear/detachment
5. Microbial keratitis – fungal or bacterial
6. Panophthalmitis/endophthalmitis, or sympathetic ophthalmitis – often requiring enucleation/evisceration

**Figure:**
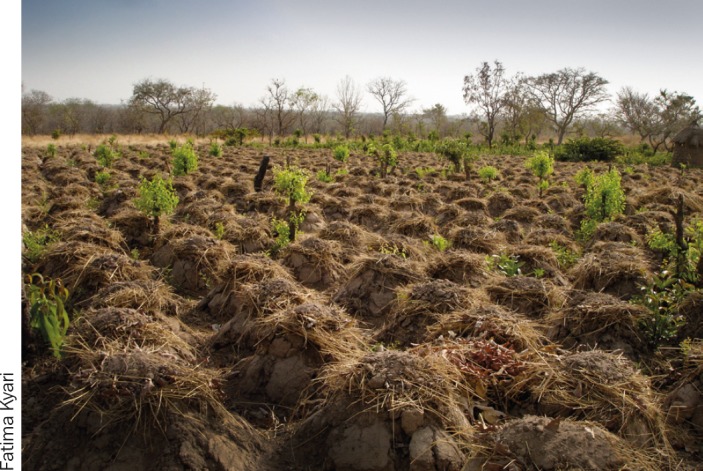
People involved in agriculture are at risk of eye injury. NIGERIA

Four decades ago, a hospital case series in Nigeria reported that 15 out of 21 patients with mycotic keratitis (71%) had a history of eye injury, 10 (66.7%) of which involved vegetative matter.^9^ One recent retrospective review of corneal ulcers/suppurative keratitis showed that the most common predisposing factor was trauma (seen in 51.3%); of these, 36/117 (30.8%) were from plant/vegetable matter.^10^

Poor **prognostic factors** for agriculture-related eye injuries are:

1. Nature of injury: worse prognosis if due to vegetative material and exacerbated by inappropriate use of traditional eye medication or steroid eye drops.
2. Severity of injury: worse if it is a penetrating injury or an injury to multiple ocular structures
3. Late presentation at a health care facility
4. Evidence of infection at the time of presentation
5. Difficulty in management and inadequate treatment options for eye injuries in health care facilities, e.g. lack of required products such as bandage contact lens, visco-elastic and fine nylon sutures; and the lack of support services for therapeutic keratoplasty, corneal repair within 24 hours and vitreo-retinal surgical facilities.

## Prevention and management

A large sector of the population is at risk of monocular blindness from agriculture-related eye injuries, so there is a need for prevention. However, there is very little evidence (from research in this area) to guide and develop appropriate messages or policy. Some possible measures include:

1. Raising public awareness and health education through television or radio programmes on eye safety or by giving health education talks in hospital/clinic waiting rooms.
2. Encouraging the use of protective eyewear by those at risk and making such eyewear available and affordable.
3. Establishing a national or state-based ocular injuries register to record incidence by type, cause and pattern of injury. This could be used to inform appropriate public policy and legislation on eye safety.
4. Working with hospitals to develop policies that will allow management of eye injuries on an emergency basis. Departments can collaborate and work out a payment schedule so that treatment/surgery can be initiated without having to wait for payment of fees by patients.
5. Lobbying government, or insurance companies directly, for health insurance to cover the treatment of eye injuries.
6. Demarcating dedicated ranches or areas of free-grazing for livestock/cattle-rearing which are separate from areas of crop farming. This will help to prevent communal clashes between farmers. This is being implemented in some communities at present.
